# Does preparation help to switch auditory attention between simultaneous voices: Effects of switch probability and prevalence of conflict

**DOI:** 10.3758/s13414-023-02841-y

**Published:** 2024-01-11

**Authors:** Amy Strivens, Iring Koch, Aureliu Lavric

**Affiliations:** 1 Institute for Psychology, RWTH Aachen University, Jägerstraße 17-19, 52066 Aachen, Germany; 2 Department of Psychology, University of Exeter, Exeter, UK

**Keywords:** Cognitive and attentional control, Attention: Selective, Audition

## Abstract

Switching auditory attention to one of two (or more) simultaneous voices incurs a substantial performance overhead. Whether/when this voice ‘switch cost’ reduces when the listener has opportunity to prepare in silence is not clear–the findings on the effect of preparation on the switch cost range from (near) null to substantial. We sought to determine which factors are crucial for encouraging preparation and detecting its effect on the switch cost in a paradigm where participants categorized the number spoken by one of two simultaneous voices; the target voice, which changed unpredictably, was specified by a visual cue depicting the target’s gender. First, we manipulated the probability of a voice switch. When 25% of trials were switches, increasing the preparation interval (50/800/1,400 ms) resulted in substantial (~50%) reduction in switch cost. No reduction was observed when 75% of trials were switches. Second, we examined the relative prevalence of low-conflict, ‘congruent’ trials (where the numbers spoken by the two voices were mapped onto the same response) and high-conflict, ‘incongruent’ trials (where the voices afforded different responses). ‘Conflict prevalence’ had a strong effect on selectivity–the incongruent–congruent difference (‘congruence effect’) was reduced in the 66%-incongruent condition relative to the 66%-congruent condition–but conflict prevalence did not discernibly interact with preparation and its effect on the switch cost. Thus, conditions where switches of target voice are relatively rare are especially conducive to preparation, possibly because attention is committed more strongly to (and/or disengaged less rapidly from) the perceptual features of target voice.

The issue of how a listener attends to a talker in the presence of other concurrent speech (the ‘cocktail party problem’) has intrigued researchers since Cherry’s (1953) original dichotic listening experiments. Following these seminal experiments, researchers have employed a number of paradigms to investigate selective auditory attention to voices. For example, the popular ‘call sign’ (or ‘coordinate response measure’) paradigm, in which each of several simultaneous voices says a sentence containing a call sign to which participants are required to attend, has been used to investigate speech intelligibility in complex environments (Eddins & Liu, 2012), awareness of the relevant voice location (Kidd et al., 2005), effects of familiarity with voices (Johnsrude et al., 2013), the temporal predictability of the target voice (Kitterick et al., 2010), effects of preparation on listener’s performance (Holmes et al., 2018), and other cocktail-party phenomena (cf. Humes et al., 2017).

In another paradigm, developed to contrast multitalker conditions with stable versus dynamically changing spatial and nonspatial parameters, Best et al. (2008) played series of four numbers simultaneously from five loudspeakers, with a light indicating the target loudspeaker. Voice identity and location were manipulated independently to be constant or vary across the four-number series, and the light cue occurred concurrently with, or in advance of, the onset of voices. When target location was constant throughout the sequence, participants’ accuracy of reporting the four-number sequence at the target location improved with every number and was generally higher than when target location switched. Performance was also better when the location cue was presented in advance, but only when the same voice was presented at the target location. Best et al. (2008) concluded that the listener attends to a voice as a perceptual object built over time from nonspatial and spatial parameters, hence the detrimental effects of switches on performance (see also Best et al., 2010).

Although the above studies by Best et al. (2008, 2010) gained valuable insights into the effects of switching spatial and nonspatial features of voices, they were primarily concerned with the role of continuity in the gradual improvement of attentional selectivity. A more recent line of research has had intentional switching of auditory attention as its primary focus. In a first study of this kind, Koch et al. (2011) have combined the cocktail-party set-up with task-switching methodology (e.g., Kiesel et al., 2010; Koch et al., 2018; Monsell, 2003). On each trial they presented dichotically two simultaneous talkers (a female and a male), each saying a single-digit number, and asked participants to categorize the number spoken by the voice specified by a visual gender cue. This paradigm was specifically designed for comparing reaction times (RTs) and accuracy for switches versus repetitions of the target voice. Both measures revealed a substantial performance detriment for switches relative to repetitions—the ‘switch cost’ (Koch et al., 2011). Importantly, because the target voice was the only aspect of the task that could change over trials (whilst all the other aspects of the task, particularly the categorization and responses, remained constant), the switch cost could be unequivocally attributed to auditory attention switching between the voices.

Task-switching research has shown that one way to reveal the contribution of intentional (top-down) control to the switch cost is to manipulate the preparation (cue-stimulus) interval (CSI) and examine whether this leads to a reduction in the switch cost. Koch and colleagues have done so and found that preparation improved the overall performance, which has been confirmed by subsequent studies using the same paradigm or other paradigms (e.g., research which combined the use of voice cueing and ‘coordinate response measure’ stimuli; Holmes et al., 2018). However, in Koch et al.’s experiments, preparation did not conclusively reduce the target voice switch cost—it appeared to do so in one experiment, but a subsequent, better controlled experiment did not find a significant reduction in the switch cost with preparation.

A series of studies by the same research group used Koch et al.’s (2011) paradigm to investigate the influence of various manipulations on the voice switch cost, including the manipulation of preparation interval. Lawo et al. (2014) cued the target voice by gender or by location and found no significant effect of preparation on the switch cost for either selection criterion. Lawo and Koch (2015) found no clear reduction in switch cost with preparation across a variety of response mappings and effectors. Seibold et al. (2018) tested the use of auditory voice cues, thus avoiding cue-stimulus changes of perceptual modality, and used only one voice per gender in the gender-cueing blocks (which could conceivably facilitate preparation for an individual voice, relative to one of several possible talkers of each gender in earlier studies). However, these changes to the paradigm still did not result in a consistent reduction in switch cost with preparation—except when the target voice alternated in predictable runs.

Previous evidence shows that preparation tends to reduce switch costs in studies of visual task switching (e.g., Meiran, 1996; Monsell & Mizon, 2006; Rogers & Monsell, 1995; Van’t Wout et al., 2013), and there is relative agreement that this effect indexes top-down control of task/attentional set (e.g., Monsell, 2015). Hence, the above voice-switching studies raise the possibility that it may be difficult (or perhaps less beneficial for performance) to ‘retune’ to another voice in advance of hearing it than to prepare for switching a (typically) visual task before task execution. This may be due to some key differences between conventional visual task switching (where switches involve changes in the relevant categorization and stimulus–response mappings) and auditory switching in cocktail-party scenarios, where all these aspects are held constant and the only aspect that can change is which voice auditory attention must select. However, similar adaptations of the task-switching paradigm, which also fixed the categorization/response aspects and examined switches of auditory attention between sound amplitude and frequency (Nolden & Koch, 2023), or between short patterns and long patterns in sequences of sounds (Nolden & Koch, 2017), and switching between the auditory and visual modalities (Lukas et al., 2010) found that preparation significantly reduced the respective switch costs.

To determine whether switches of attention between simultaneous voices are indeed less amenable to preparation, Monsell et al. (2019) modified the gender-cueing paradigm developed by Koch et al. (2011) to optimize the conditions for encouraging preparation and for detecting its benefits. They reduced the probability of a voice switch (from 50% to 33%^1^), because lower switch probabilities have resulted in steeper reductions in task switch costs with preparation (e.g., Mayr et al., 2013; Monsell & Mizon, 2006). They also reduced the proportion of response-congruent trials (where both voices required the same response) from 50% to 20% and analyzed only the response-incongruent trials, and limited the number of voices per gender to one, presenting the voices diotically (centrally).^2^ Monsell and colleagues also reasoned that preparation may be (more) effective when voices are familiar and when their onsets are not entirely simultaneous, hence they manipulated both variables—but found substantial (~40%) reduction in switch costs with preparation in all conditions—irrespective of voice familiarity and simultaneity.

Monsell et al.’s (2019) experiments demonstrated that, provided the set of conditions described above, the benefit of preparation for switching attention between voices can be revealed. This implies that top-down, intentional control can be engaged effectively to reduce the voice switch cost provided that these conditions are met. But which of the above conditions are *essential* for encouraging and detecting effective preparation? The current study focuses on two of the factors that were different in Monsell et al. (2019) relative to earlier voice switching studies: the probability of a switch in the target voice and the proportion of response-congruent trials (where the numbers spoken by voices afford the same response). Our reasons for choosing these two factors are as follows. As already mentioned above, Monsell et al.’s (2019) results showed that familiarity and simultaneity did not materially influence the reduction in switch cost with preparation. Seibold et al. (2018) showed that the use of a single voice per gender was not sufficient to produce/detect an effect of preparation on the switch cost. We therefore assume that these variables play at most a modest role in the elicitation and detection of a reduction in switch cost with preparation. In contrast, there is substantial evidence in the task-switching literature using visual stimuli (see below for details) that switch probability has an effect both on the switch cost and on its reduction with preparation. Yet, for reasons already discussed, (preparing for) a switch of the target voice in the cocktail party scenario is not the same as (preparing for) performing another task. Hence, it is important to examine the influence of the switch probability on the reduction in the cost of switching auditory attention between voices.

With regard to the proportion of response-congruent trials (henceforth referred to as *proportion congruent*), we are not aware of any previous evidence concerning its effect on the reduction in switch cost with preparation. This in itself is a motivation, but there are at least three further reasons to investigate the role of this variable. First, as noted by Monsell et al. (2019), a relatively high (50%) proportion of trials where the attentional selection of the target voice is not strictly necessary (because the response to the nontarget voice would not result in an error) may well discourage participants (at least to some extent, or on some trials) from engaging in effortful preparation. Second, there is considerable evidence from task-switching studies (as will be reviewed later), that a lower proportion congruent results in reduced interference during response-selection–indicative of greater engagement of top-down control (e.g., Bugg & Braver, 2016; Bugg & Crump, 2012). Third, when the switch/repetition is limited to the perceptual (attentional selection) aspect of the task, as it is for voice-switching (Koch et al., 2011), preparatory attention may result in extra benefits. Since the only source of response interference is the nontarget voice, inasmuch as preparation can reduce the encoding of what it says, this should reduce interference at the response stage, which may be reflected in a reduced difference between response-congruent trials and the response-incongruent trials (*congruence effect*) and, possibly, a smaller switch cost.

Thus, the primary motivation for the current research is two-fold. First, it aims to bridge the gap between the task-set control and talker selection literatures, by determining whether a variable that has a major effect on the task switch cost and its modulation by preparation, has similar effects in the multitalker setting. Hence, Experiment 1 manipulated the probability of a switch of the target voice to examine its influence on the switch cost and its reduction with preparation. Second, it asks whether the effect of preparation on the switch cost is influenced by ‘conflict prevalence’—something that hitherto has not been investigated in either the task switching or the multitalker literatures. To this end, Experiment 2 manipulated the proportion congruent to explore its influence on the congruence effect, the switch cost, and especially the reduction in switch cost with preparation.

## Experiment 1

As already mentioned, task-switching studies have documented the influence of switch probability on the task-switch cost and its reduction with preparation. Monsell and Mizon (2006) and Mayr et al. (2013) reported a steep reduction in switch cost with preparation when there were 25% switches; in the 50%-switch condition, this reduction was shallower (but remained significant), whereas in the 75%-switch condition, it was no longer detectable. Similar results were obtained by Kikumoto et al. (2016), who contrasted the switch probabilities of 33% and 66%. In the current experiment, we manipulated both switch probability (25% vs. 75%) and the preparation interval (CSI) while keeping other relevant design parameters the same as in Monsell et al. (2019). If switch probability indeed plays a key role in encouraging and/or detecting the benefits of advance ‘retuning’ to the target voice, we expect a steeper reduction in switch cost with preparation in the 25%-switch condition than in the 75%-switch condition.

### Method

#### Participants

The target sample size was set to 32 in accordance with design counterbalancing constraints (it had to be a multiple of 16) and statistical power considerations (see below for details on both). A total of 34 participants recruited via Prolific (www.prolific.co) provided informed consent to participate in the two-session experiment, whose procedure was approved by the RWTH Aachen University, Faculty 7 (Arts & Humanities) Ethics Committee. The data from two participants were excluded due to the high error rate (see below). Of the remaining 32 participants whose data were analyzed, 31 had a mean age of 31.8 years (*SD* = 13, range: 18–60), and one participant did not disclose their age. There were 22 females and nine males; one participant did not disclose their gender. Because the stimuli were English words spoken in conditions of perceptual (energetic) masking from another speech stream, it was important that participants had high (native-like) English comprehension proficiency. Hence, we required participants to have reported in Prolific that they were native English speakers and that they resided in a predominantly English-speaking country at the time of testing, including UK, USA, Canada, and Australia.

### Design

The experiment had a 2 (switch vs. repetition of the target voice) × 2 (switch probability, 25% vs. 75%, tested in separate sessions) × 4 (CSI) repeated-measures design. The dependent variables were reaction time (RT; ms) and the error rate (%).

#### Statistical power considerations

Our approach to determining the number of participants for achieving optimal sensitivity (statistical power) was threefold. First, we examined the number of participants required to detect a preparation effect in our low switch probability condition, by relying on our recent analysis of the effect sizes of preparation effects in 10 published experiments conducted in the Exeter laboratory (reported in Monsell et al., 2019), in nine of which task switches were relatively rare (33%). This analysis found the reduction in switch cost with preparation to have a large effect size and concluded that 10 participants were needed to achieve power ≥0.8, and 12 participants were needed to achieve power ≥0.9. Our sample of 32 should therefore ensure more than adequate sensitivity for detecting the predicted reduction in switch cost with preparation in the 25%-switch condition. Second, with regard to detecting the influence of switch probability on the preparation effect, we examined the only within-participants experiment we could find, which tested for and detected a significant interaction between switch/repeat, CSI, and switch probability (Siqi-Liu & Egner, 2020, Experiment 1). Our number of observations in the smallest cell of this interaction within a participant (32) and across all participants (1,024) is comparable with (somewhat larger than) the number of observations in the smallest cell in Siqi-Liu and Egner’s (2020) experiment (27 and 1,080, respectively).

Finally, we also considered the sensitivity to the two-way interaction between switch/repeat and switch probability, which has been tested in a number of task-switching studies. We looked at studies which found a robust two-way interaction in experimental conditions that map onto the conditions in our experiment and excluded observations associated with other experimental conditions/manipulations. Liu and Yeung (2020, Experiment 1) had 560 observations in the smallest cell of this interaction in total (over all participants), 28 per participant; Dreisbach and Haider (2006) had a total of 600 observations (25 per participant); Siqi-Liu and Egner (2020, Experiment 4) had 2,624 (32 per participant); and Bejjani et al. (2021) had 5,376 (64 per participant). Our experiment, with a total of 4,096 observations in the smallest cell of our analysis (128 per participant), is at the higher end of these observations counts. We conclude that the present study is more than adequately powered to detect reduction in switch cost with preparation in the low switch probability condition, as well as the influence of switch probability on the switch cost and its reduction with preparation.

#### Task and materials

The experiment was conducted using Gorilla Experiment Builder (www.gorilla.sc). The task was to listen to one of two simultaneous talkers (a male and a female), each saying a number, and categorize the number spoken by the target voice, specified by a prestimulus picture cue, as <5 vs. >5 via a computer key press. The voice stimuli were recordings of two males and two females, each saying one of eight numbers (referring to the digits 1–9, excluding 5). One female speaker was recorded by the RWTH Aachen Institute of Technical Acoustics in an anechoic chamber (Loh & Fels, 2020); the remaining speakers were recorded in nonspecialist conditions whilst ensuring that recordings contained no background noises or echoes.

Several recordings of each number were made for each voice to optimize the preparation of voice stimuli. For each of the four male–female pairs, all combinations of the numbers spoken by the two voices were used to create two-talker compounds, except the eight combinations where the two voices said the same number. The durations of all individual utterances were set to 600 ms, with the first vowel starting at approximately the same point in the different recordings to achieve uniform energetic masking in compounds. The fundamental frequencies were selected and/or adjusted to minimize the within-speaker variability whilst keeping a reasonably consistent range across the voice pairs. The sound intensities of the utterances were edited to have similar subjective volume as judged by two listeners. The four voice pairs were each allocated to eight participants, ensuring that each participant encountered only one pair (one male speaker and one female speaker, presented centrally/diotically) throughout both testing sessions—one session per switch probability condition.

**Fig. 1:**
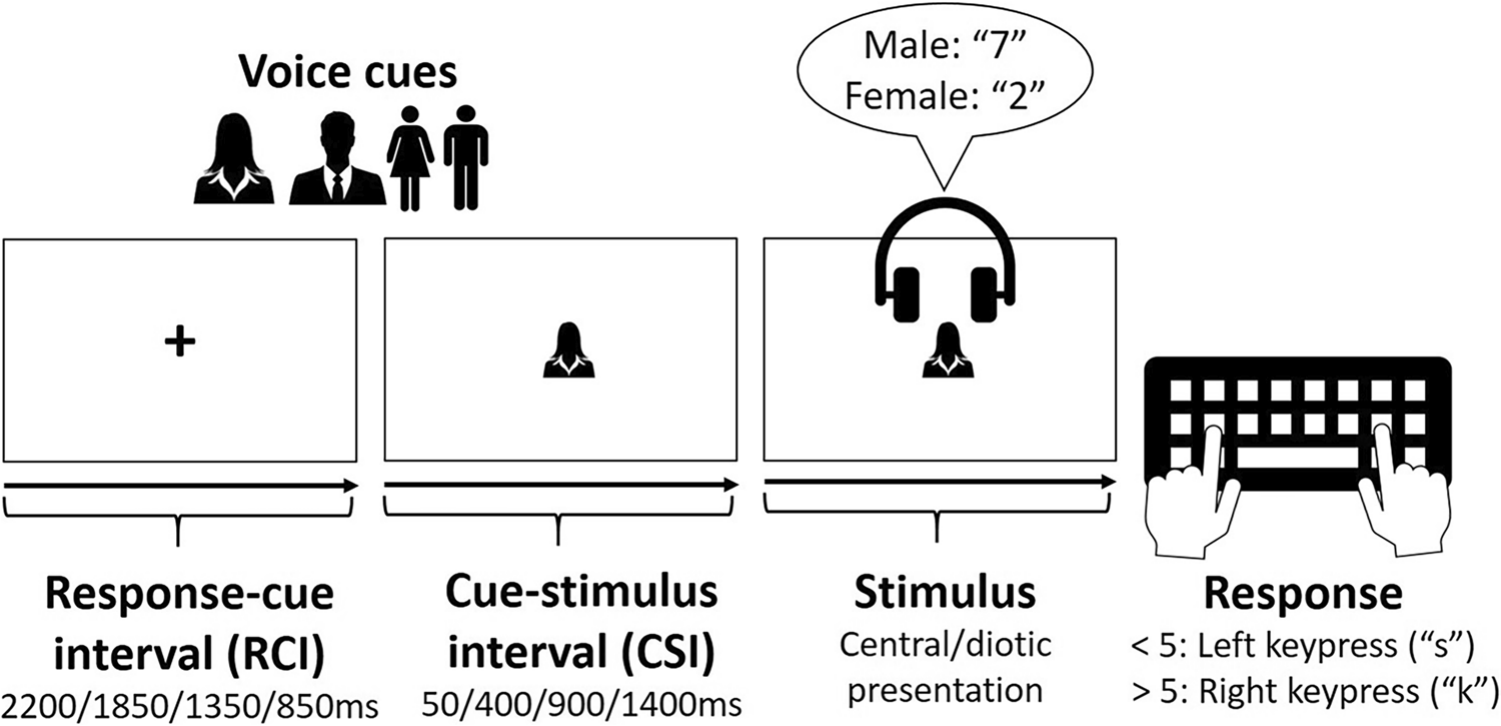
Voice cues and the time-course of one trial

One of four semantically transparent pictorial cues was displayed centrally to specify the gender of the target voice on each trial (a silhouette and a full-body body icon for each gender; see Fig. 1). The silhouette and icon were always alternated from one trial to another (after randomly picking one of them to start a block of trials), to avoid immediate cue repetition, and thus unconfound the switch/repetition of target voice from the switch/repetition of the cue (cf. Monsell & Mizon, 2006; Monsell et al., 2019). Cue dimensions in pixels (in parentheses, in mm, on a laptop with a 14.2-in. screen) were male icon, 70 × 155 (15 × 36); male silhouette, 125 × 115 (29 × 27); female icon, 82 × 154 (19 × 36); female silhouette, 105 × 115 (25 × 27). The onset of the cue preceded the voice compound by one of four cue-stimulus intervals (CSIs): 50/400/900/1400ms. The cue remained on the screen throughout the CSI and poststimulus onset until the participant responded. The CSI durations entered in Gorilla were 50 ms shorter to allow for a 50-ms delay in playing sound files (based on our pilot testing using Gorilla). On each trial the cue was preceded by a fixation cross whose duration (the response-cue interval [RCI]) varied inversely to that of the cue (2,200/1,850/1,350/850 ms) ensuring a constant response–stimulus interval of 2,250 ms for all four CSIs and thus unconfounding the time available for preparation from the time available for the decay/dissipation of ‘attentional inertia’ (e.g., Longman et al., 2014, 2017) from the previous trial. CSI and RCI were constant within a block but varied over blocks (see below). Following the onset of the stimulus (the two simultaneous speech streams), the participant had 3,000 ms to press the ‘s’ key when the number spoken by the target voice was <5 or the ‘k’ key if it was >5. An incorrect key press led to ‘Error’ being displayed centrally for 3,000 ms (practice blocks) or 2,000 ms (main blocks). Failure to respond before the response deadline led to ‘No response’ being displayed centrally for 3,000 ms.

A script was developed to create two unique randomized sequences of trials for every participant—one for each session (i.e., switch probability condition)—which were subsequently used in Gorilla. In what follows, we outline the structural constraints on the trial sequences used in the 25%-switch condition (the 75%-switch condition used structurally equivalent sequences, except swapping the constraints that applied to switches vs. repeats, e.g., the 1 switch:3 repeat ratio became 3:1). The trial sequence for the 25%-switch condition consisted of four subsequences—one for each CSI. Each subsequence contained 160 trials, of which 128 (80%) were response-incongruent and 32 (20%) were response-congruent. A quarter of the trials (32 response-incongruent and eight response-congruent) were switch trials and ¾ were repeat trials (96 and 24, respectively). On half of the switch trials and on half of the repeat trials the target voice was that of the female speaker, and on the remaining halves of switch and repeat trials the target voice that of the male speaker—this was also true separately for incongruent and congruent trials. Subdividing further, half of the combinations involving each target voice (e.g. incongruent switch male) had ‘s’ as the correct response and half had ‘k’ as the correct response. This meant that the combinations of CSI × switch/repeat × response congruence × voice gender × response category were perfectly balanced for each participant and each session. The sequencing script also balanced (where possible) or randomized (where balancing was not possible) the combinations of the numbers spoken by the target and nontarget voices over the combinations of switch/repeat × CSI × voice gender (for details, see Appendix A). The numbers spoken on each trial by the two voices never repeated any of the numbers spoken on the previous trial.

The above four 160-trial subsequences (one for each CSI) were each divided for the testing purposes into two blocks of 80 trials and interdigitated, by including the first block of all CSIs, then the second block of all CSIs (whilst preserving the same order of CSIs in the second half of the session as in the first half; e.g., CSI = 50-Block1, CSI = 900-Block1, CSI = 400-Block1, CSI = 1,400-Block1, CSI = 50-Block2, CSI = 900-Block2, CSI = 400-Block2, CSI = 1,400-Block2. There were eight such CSI orders—one for every four participants; the order of CSIs for a given participant was the same in the two switch probability conditions (testing sessions). The CSI counterbalancing ensured that, across participants, the four CSIs occurred equally in different positions during the session, and that one of the two shortest CSIs (50 ms or 400 ms) was always followed by one of the two longest CSIs (900 ms or 1,400 ms) and vice versa.

#### Procedure

The experiment consisted of two sessions separated by at least 24 hrs, each lasting ~1 hr. Each session was allocated to a probability condition (25% or 75%), with the order of conditions over sessions counterbalanced across participants in combination with the counterbalancing of the eight CSI orders (see above), resulting in a counterbalancing group of 16 participants. Before each session, the headphone check by Milne et al. (2021), available as an open-access material on Gorilla.sc, was employed to ensure that participants were using headphones whose sound quality was adequate for the requirements of the experiment. Following the headphone check (and before the main part of the experiment) there were two practice phases. The first phase was designed to familiarize participants with the two voices (which they would hear throughout the entire experiment), the associated cues and the categorization task (including the category–response mappings). This practice phase consisted of three 16-trial blocks during which participants heard one voice on each trial saying a number (preceded by an icon or silhouette cue at CSI = 900 ms): in the first block they heard only the male voice, in the second only the female voice, and in the third the two voices alternated randomly. Participants had to categorize the number and respond with a key press (see above). This was followed by the second practice phase, which consisted of four 25-trial practice blocks (one for each CSI) where both voices were presented simultaneously; the temporal structure of each trial (see above, Task and Materials, and Fig. 1) and the proportion of switch trials were the same as in the main part of the session that followed.

Following the two practice phases, the instructions for the main part of the experiment were displayed. Participants were instructed that the task in the main part would be the same as in the second practice phase, but in longer blocks. They were also informed about the probability of a switch of the target voice in that session (excerpt from instruction from the 25%-switch session): ‘The current session has 25% switch trials. Therefore, in the following practice blocks and main blocks you will notice that the voice to attend to will remain the same more often than it changes.’ Participants were also informed of a performance-related monetary bonus (see below) and the main experiment began. It consisted of eight blocks of 81 trials (648 trials in total): Each of these blocks contained half of a sequence of trials for one of the CSIs (80 trials), described above, plus a response-incongruent start-up trial subsequently excluded from the analysis. The voice on the start-up trial was selected depending on the voice and switch/repeat condition on the subsequent (to be analyzed) trial, whereas the response category and the spoken numbers were selected randomly.

#### Performance-related monetary bonus

To keep participants engaged, a performance score based on the overall RT and number of errors was calculated for each block, starting from the second practice phase, using the formula: mean RT/10 + number of errors × 5. After completing the second practice phase, participants were informed that from then on they would receive a bonus of 30 pence (GPB 0.3) each time the score for that block was better than the target score—the mean score for the blocks with the same CSI (including the relevant block from the second practice phase). Following each block, participants were displayed: their score for that block, whether it exceeded the target score (earning them 30 pence) and the new target score to beat. At the end of the session the number of bonuses earned during that session was displayed together with their total monetary value.

### Results

We excluded trials reported by Gorilla to have had a loading delay >10 s between the response and the fixation cross of the following trial, as well as trials where the CSI was more than 70-ms longer than intended, or where the fixation display (response-cue interval) was more than 20-ms longer than intended—such issues could arise because of fluctuations in the quality of the internet connection. A total of seven trials had to be excluded for these reasons over all participants. The data from participants whose error rate exceeded the 3 standard deviations of the entire sample were excluded and replaced (two participants).

Following Monsell et al. (2019), we restricted the analysis to response-incongruent trials (80%) from the main part of the experiment (excluding practice) on the grounds that only on incongruent trials participants had to select the target voice in order to respond above chance. We excluded the start-up trial from each block and trials following errors, and trials where RT <200 ms from both RT and error analyses (one trial where the RT of 203 ms came very close to this criterion was also excluded). Following reports from participants that sometimes they could not hear the voices on the start-up trial, the following trial (the second trial of each block) also had to be excluded because it could not be classified with confidence as a switch or repetition of the target voice. Error trials were excluded from RT analyses. The mean RTs and error rates of all participants were then submitted to repeated-measures analyses of variance (ANOVAs), with factors SwitchProb (switch probability with two levels), Switch/Repeat (2), and CSI (4); the Huynh–Feldt correction was used for violations of sphericity when these occurred (but uncorrected degrees of freedom [*df*s] are reported).

**Fig. 2:**
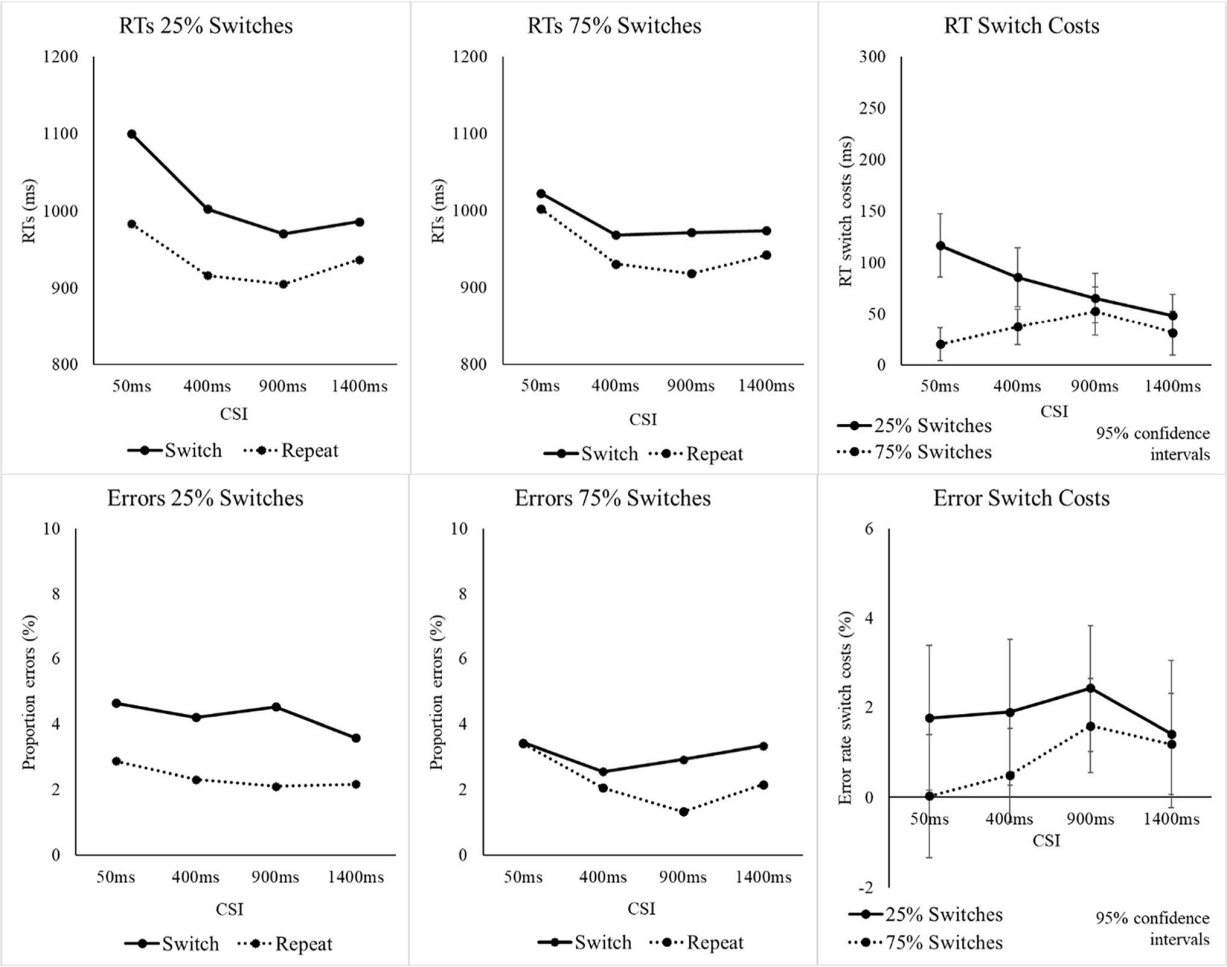
**R**T, errors, and switch costs in Experiment 1 as a function of switch probability, CSI, and switch versus repetition of the target voice. *Note,* Error bars show 95% confidence intervals around the mean switch cost

The descriptive statistics are provided in Fig. 2 and a full table with the inferential statistics is available in Appendix B. The omnibus ANOVA for RTs revealed a significant main effect of CSI, *F*(3, 93) *=* 14.16, *p* < .001, η_p_^2^ = .314, reflecting an improvement in overall performance as CSI increased, and a significant main effect of Switch/Repeat, *F*(1, 31) = 84.64, *p* < .001, η_p_^2^ = .732—the switch cost—which was larger in the 25%-switch condition (as indicated by the significant Switch/Repeat × SwitchProb interaction), *F*(1, 31) = 16.65, *p* < .001, η_p_^2^ = .349, and which reduced with preparation–the preparation effect (significant Switch/Repeat × CSI interaction), *F*(3, 93) = 3.17, *p =* .028, η_p_^2^ = .093. Crucially, the significant three-way interaction between Switch/Repeat, CSI, and SwitchProb, *F*(3, 93) = 7.15, *p* < .001, η_p_^2^ = .187, revealed a steeper preparation effect in the 25%-switch condition (where a follow-up ANOVA revealed a significant CSI × Switch/Repeat interaction), *F*(3, 93) = 9.35, *p* < .001, η_p_^2^ = .232, than in the 75%-switch condition (where the two-way interaction was not significant, *F* = 1.74). However, despite evidence for the preparation effect in the 25% switch condition, the switch cost was not eliminated by preparation, leaving a significant switch cost at the longest CSI (1,400 ms), *F*(1, 31) = 21.92, *p* < .001, η_p_^2^ = .414.

The omnibus ANOVA for the error rate (see Appendix B for the full inferential statistics) found only a significant main effect of Switch/Repeat, *F*(1, 31) = 16.05, *p* < .001, η_p_^2^ = .341—the error switch cost. Although the switch cost was larger for the 25%-switch condition, neither the interaction between SwitchProb and SwitchRepeat, *F*(1, 31) = 2.81, *p =* .104, η_p_^2^ = .083, nor the three-way interaction of these factors with CSI, *F* < 1, reached significance.

Although in all the analyses above we included only the 80% incongruent trials (on the grounds that on congruent trials a correct response can be made even without attending to the target voice), we have also conducted an ANOVA that tested for a potential effect of switch probability on the magnitude of the congruence effect,^3^ with the factors SwitchProb and Congruence. Since there were only 20% congruent trials, to ensure sufficient observations in the congruent cells, we pooled the trials over CSIs (and did not include CSI as a factor). For RTs, the congruence effects were very small both in the 75%-switch condition (5 ms, *SE* = 5.73) and in the 25%-switch condition (1 ms, *SE* = 6.80), resulting in a nonsignificant main effect of Congruence, *F* < 1, and a nonsignificant SwitchProb × Congruence interaction, *F* < 1. For errors, the congruence effects of 0.72% (*SE* = 0.29) in the 75%-switch condition and 0.73% in the 25%-switch condition (*SE* = 0.35) were reflected in a significant main effect of Congruence, *F*(1, 31) = 8.06, *p =* .008, η_p_^2^ = .206, and a nonsignificant SwitchProb × Congruence interaction, *F* < 1.

### Discussion

The results from Experiment 1 show that in the 25%-switch condition the RT switch cost was reduced substantially (~50%) and significantly by preparation; this reduction was significantly steeper than in the 75%-switch condition, where preparation did not reduce the switch cost even numerically. These results support our prediction that the probability of a change in the target voice would influence both the auditory attention switch cost and its reduction with preparation. To the best of our knowledge this is the first investigation of the influence of switch probability in a paradigm where only the perceptual component of the task set switches, without a concurrent switch in other aspects of the task-set, such as the stimulus–response mappings. It is also the first time that this effect has been demonstrated for selective auditory attention to a voice in the cocktail party setting. There are several types of account of the effect of switch probability on the switch cost and its reduction with preparation. They will be considered in the General Discussion.

## Experiment 2

As we mentioned in the Introduction, the effect of the relative proportion of response-congruent versus response-incongruent trials (which we have referred to as proportion congruent) on the reduction in the voice switch cost with preparation has not been examined thus far in either voice-switching or task-switching (to the best of our knowledge). There are reasons to expect that reducing the proportion congruent (e.g., from 50% to 20%; Monsell et al., 2019) may encourage participants to be more selective. One measure of selectivity is the *congruence effect*—the difference in performance between response-congruent and response-incongruent trials (e.g., Kiesel et al., 2010)—which has been shown to be highly sensitive to manipulations of the proportion congruent; reducing the latter diminishes the congruence effect (Braverman & Meiran, 2015; Bugg & Braver, 2016; Schneider, 2015). It has been proposed that a lower proportion congruent (meaning a higher proportion of incongruent trials) increases response conflict leading to more top-down control being applied, which, in turn, increases the selectivity of attention towards the target stimulus attribute (perceptual attribute and/or S-R mapping) and away from the nontarget stimulus attribute (Bugg & Crump, 2012). If reducing the proportion congruent leads to ramping up in top-down control, one might expect it to also reduce the switch cost–and there is some evidence from analyses of error switch costs that this is indeed the case (Bugg & Braver, 2016; Schneider, 2015).

Of some relevance for the present investigation is the evidence that a longer preparation interval can reduce the congruence effect (e.g., Meiran et al., 2000; Monsell & Mizon, 2006), though other studies do not find such an effect (e.g., Allport et al., 1994; Rogers & Monsell, 1995). However, none of the studies above have examined whether the effects of preparation on the congruence effect, or on the switch cost, were influenced by the proportion congruent. This is what the present experiment investigates in the context of the cocktail-party setting, whilst keeping the switch probability low (33% as in Monsell et al., 2019) to maximize the effects of preparation on the switch cost.

### Method

#### Participants

A total of 48 participants recruited using Prolific (using the same inclusion criteria as in Experiment 1; see above) provided informed consent to participate in the two-session experiment whose procedure was approved by the RWTH Aachen University, Faculty 7 (Arts & Humanities) Ethics Committee. The data from one participant was excluded due to the high error rate (see below). The remaining 47 participants had a mean age of 35.5 years (*SD* = 12.4, range: 18–71); 23 were females, 23 males, and one person selected ‘Other.’

#### Design

The experiment had a 2 (switch vs. repetition of the target voice) × 2 (proportion congruent, 33% vs. 66%, tested in separate sessions) × 2 (congruent vs. incongruent trial type) × 2 (CSI) repeated-measures design. The dependent variables were RT (ms) and the error rate (%).

#### Statistical power considerations

We are not aware of studies that have tested the interaction between CSI, switch/repeat, and proportion congruent. Therefore, we used the recommendations of Brysbaert and Stevens (2018) for power in a repeated-measures design in order to ensure we had sufficient power to detect a small to medium effect. They recommended that a repeated-measures design experiment should have at least 1,600 observations in the smallest cell of the analysis to ensure adequate power for detecting a medium-size effect. In the 33% congruence condition, we have 48 congruent switch trials of each CSI (in the 66% congruence condition, this is the case for incongruent switch trials of each CSI). Across 47 participants, this amounts to a total of 2,256 observations in the smallest cell of our analysis. Hence, according to the above criterion, the experiment is at least adequately powered.

#### Materials, task, and procedure

The materials, online testing platform, task and procedure were the same as in Experiment 1, except for the following differences. CSI was manipulated over two (rather than four) levels: 50/1,000 ms. The response-cue interval (RCI) varied inversely (2,165/1,215 ms) ensuring a constant response–stimulus interval of 2,265 ms.

The script for creating two unique randomized trial sequences for each participant (one for each proportion congruent session) had to be adjusted to account for the reduced number of CSIs, and a different switch probability, as well as to permit the manipulation of proportion congruent. In what follows, we outline the structural constraints that had to be satisfied by sequences used in the 33%-congruent condition (the 66%-congruent condition used structurally equivalent sequences, except swapping the constraints that applied to congruent vs. incongruent trials, e.g., the 1 congruent:2 incongruent ratio became 2 congruent:1 incongruent). The trial sequence for the 33%-congruent condition consisted of two subsequences—one for each CSI. Each subsequence contained 432 trials, of which 288 (66%) were repetition trials and 144 (33%) were switch trials. A third of the trials (96 repeat trials and 48 switch trials) were congruent and 2/3 were incongruent trials (192 and 96, respectively). On half of the congruent trials and on half of the incongruent trials the target voice was that of the female speaker, and on the remaining halves of congruent and incongruent trials the target voice was that of the male speaker. This was also true for repeat and switch trials taken separately. Subdividing further, half of the combinations involving each target voice (e.g., incongruent switch male) had ‘s’ as the correct response and half had ‘k’ as the correct response. This meant that the combinations of CSI × switch/repeat × response congruence × voice gender × response category were perfectly balanced for each participant. The sequencing script also balanced (where possible) or randomized (where balancing was not possible) the allocations of the combinations of numbers spoken by the target and nontarget voices over the combinations of CSI × switch/repeat × voice gender (for details, see Appendix C). As in Experiment 1, the numbers spoken on each trial by the two voices never repeated any of the numbers spoken on the previous trial.

The above two 432-trial subsequences (one for each CSI) were each divided (for the testing purposes) into blocks of 72 trials and interdigitated, by including the first block of both CSIs, then the second block of both CSIs (whilst maintaining the order of CSIs in the second half of this sequence; e.g., CSI = 50-Block1, CSI = 1,000-Block1, CSI = 50-Block2, CSI = 1,000-Block2, CSI = 50-Block3, CSI = 1,000-Block3). The order of the CSIs was counterbalanced (24 participants were presented with the 50-ms CSI first and 24 with the 1,000-ms CSI^4^); the order of CSIs for a given participant was the same in the two proportion congruent conditions (testing sessions).

### Results

The same criteria as in Experiment 1 were used to exclude trials affected by delays caused by Gorilla and/or the internet connection (53 trials over all participants). One participant’s data was excluded because their error rate exceeded 3 standard deviations of the entire sample. As in Experiment 1, we excluded the first two start-up trials from each block, trials following errors, and trials where RT < 200 ms from both RT and error analyses. Error trials were excluded from RT analyses. The mean RTs and error rates of all participants were then submitted to repeated-measures ANOVAs, with factors PropCong (proportion congruent with 2 levels), Switch/Repeat (2), Congruence (2), and CSI (2); the Huynh–Feldt correction was used for violations of sphericity when these occurred (but uncorrected *df*s are reported).

**Fig. 3:**
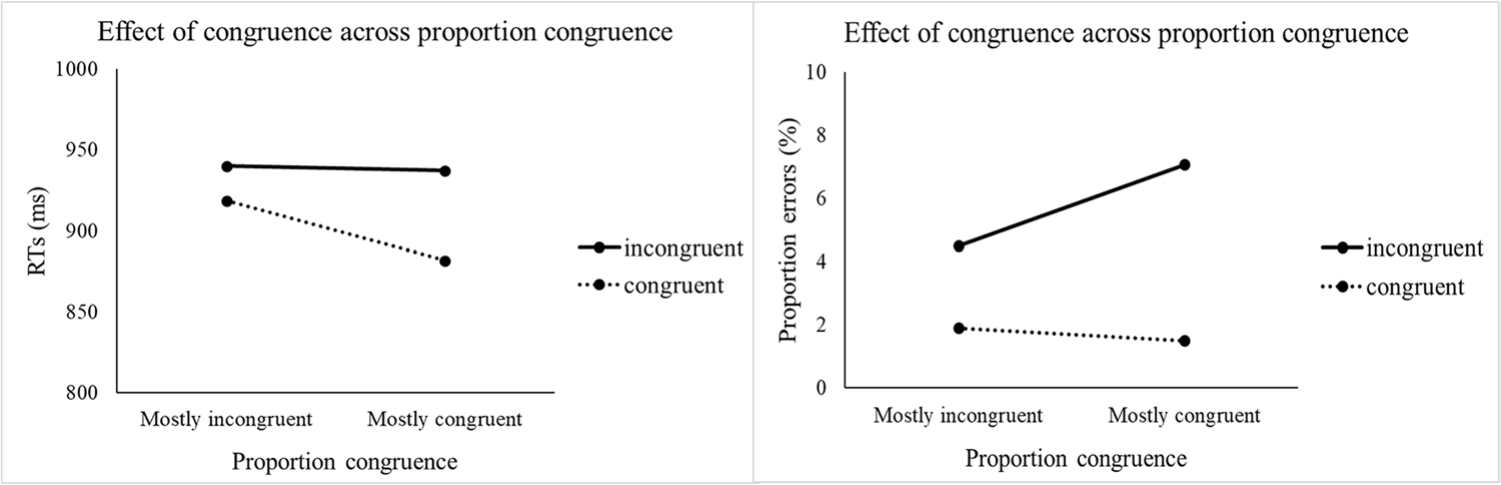
**G**raphs of RTs (left) and error rates (right) in congruent and incongruent trials as a function of PropCong

**Fig. 4:**
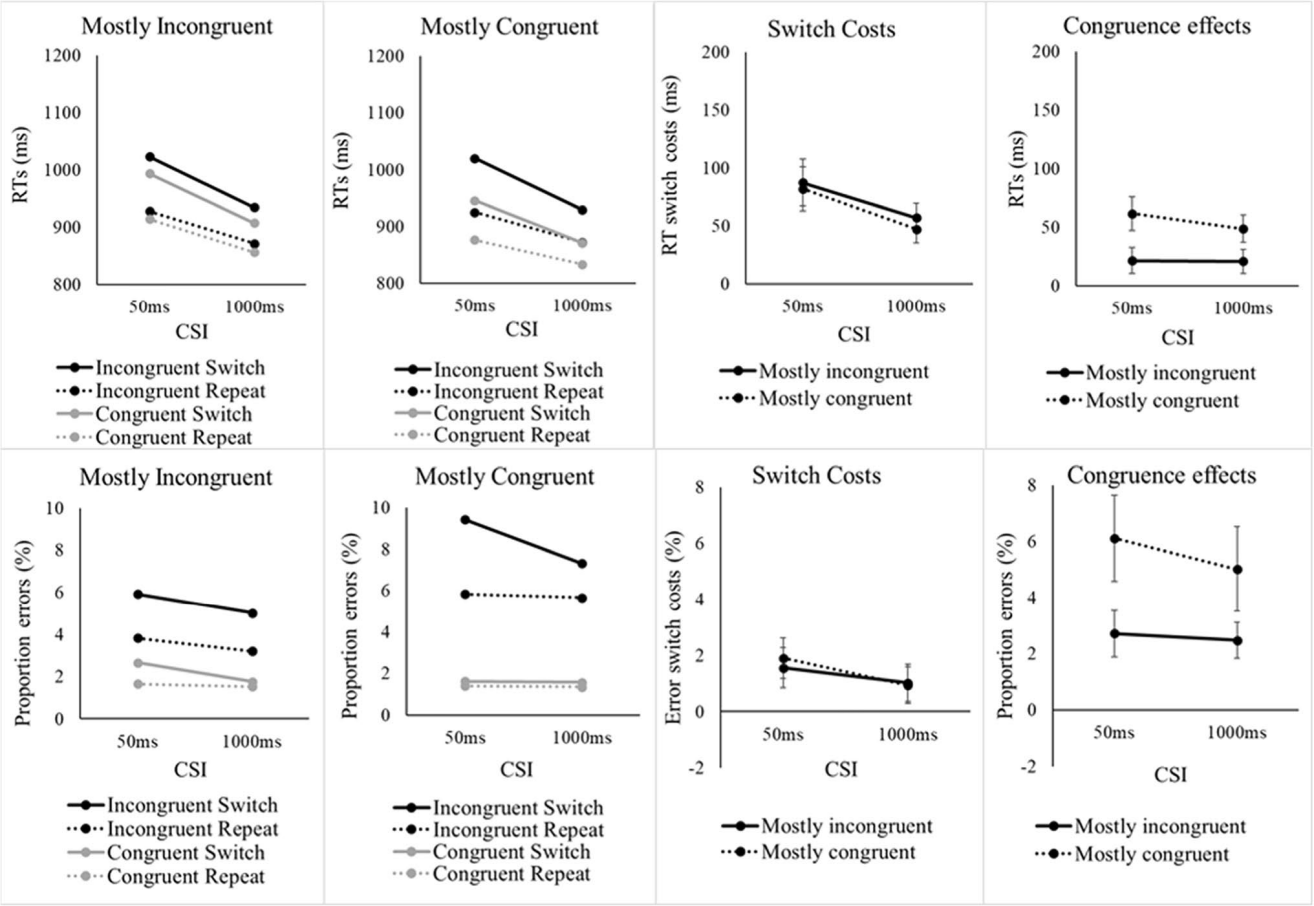
**RT** and errors (left) in Experiment 2 as a function of PropCong, CSI, congruence, and switch versus repetition of the target voice. Switch costs and congruence effects (right) in Experiment 2 as a function of PropCong and CSI. *Note* Error bars show 95% confidence intervals around the mean switch cost

The descriptive statistics are provided in Figs. 3 and 4 and a full table with the inferential statistics is available in Appendix D. The omnibus ANOVA of PropCong, CSI, Congruence, and Switch/Repeat for RTs showed a significant main effect of CSI, *F*(1, 46) = 54.32, *p* < .001, η_p_^2^ = .541, reflecting faster responses with a longer CSI. There was also a significant main effect of Switch/Repeat, *F*(1, 46) = 235.09, *p* < .001, η_p_^2^ = .836, reflecting a substantial switch cost, and a significant Switch/Repeat × CSI interaction, *F*(1, 46) = 17.19, *p* < .001, η_p_^2^ = .272, reflecting a significant reduction in switch cost with preparation. The interaction between Switch/Repeat and PropCong was not significant, *F*(1, 46) = 2.07, *p* = .157.

The significant main effect of Congruence, *F*(1, 46) = 70.61, *p* < .001, η_p_^2^ = .606, reflected poorer performance on incongruent trials than on congruent trials. Congruence interacted significantly with PropCong, *F*(1, 46) = 44.13, *p* < .001, η_p_^2^ = .490, reflecting, as in previous studies which manipulated the relative proportion of congruent/incongruent trials, a larger congruence effect in the mostly-congruent condition than in the mostly incongruent condition. There was also a significant interaction between Congruence and Switch/Repeat, *F*(1, 46) = 14.85, *p* < .001, η_p_^2^ = .244, reflecting a larger congruence effect for voice switches than for voice repetitions. The interaction between Congruence and CSI was not significant, *F*(1, 46) = 2.48, *p* = .122. Crucially the three-way interactions between CSI, Congruence and Switch/Repeat, *F* < 1, and between CSI, PropCong, and Switch/Repeat, *F* < 1, were not significant, indicating that neither Congruence nor PropCong had a detectable impact on the reduction in switch cost with preparation. The four-way interaction between CSI, PropCong, Congruence, and Switch/Repeat was also not significant, *F* < 1.

As mentioned in the Introduction, Monsell et al. (2019) suggested that having a large proportion of congruent trials can reduce one’s willingness to engage in effortful preparation. To examine whether this was indeed the case, we tested the reduction in switch cost with preparation effect separately for the two levels of PropCong, with a focus on the mostly congruent (66%) condition. The Switch/Repeat × CSI interaction was highly significant when most of the trials were congruent (66% congruent), *F*(1, 46) = 13.46, *p* < .001, η_p_^2^ = .226 (it was also significant when most of the trials were incongruent), *F*(1, 46) = 13.80, *p* < .001, η_p_^2^ = .231. Therefore, it appears that the high proportion of congruent trials did not deter participants from preparing for a switch of the target voice.

The omnibus error rates ANOVA revealed the same significant main effects of CSI, *F*(1, 46) = 11.34, *p* = .002, η_p_^2^ = 0.198, Congruence, *F*(1, 46) = 72.65, *p* < .001, η_p_^2^ = .612, and Switch/Repeat, *F*(1, 46) = 68.00, *p* < .001, η_p_^2^ = .596, as the RT ANOVA. The interactions that were significant in the RT ANOVA were also significant here: CSI × Switch/Repeat, *F*(1, 46) = 6.88, *p* = .012, η_p_^2^ = .130; Congruence × Switch/Repeat, *F*(1, 46) = 29.34, *p* < .001, ηp^2^ = .389; and Congruence × PropCong, *F*(1, 46) = 23.54, *p* < .001, η_p_^2^ = .339, all reflecting effects in the same direction as RTs. The only difference relative to the RT ANOVA was the additional significant main effect of PropCong for errors, *F*(1, 46) = 9.06, *p =* .004, η_p_^2^ = .165, with higher overall error rates in the mostly congruent condition than mostly incongruent condition.

### Discussion

Based on previous task-switching studies, we expected that a lower proportion of congruent trials will result in a ramping up of top-down control, leading to reduced interference from the nontarget voice, which should result in a smaller congruence effect and possibly a smaller switch cost. More importantly, we asked whether the adjustment in top-down attentional selectivity may start early in the trial, during the preparation interval, leading to a steeper reduction in switch cost. The results reveal a congruence effect that is clearly influenced by the proportion congruent, with a much smaller congruence effect when 33% of trials were congruent that when 66% of the trials were congruent. The switch cost and its reduction with preparation was significant for both proportions of congruent trials, with no discernible difference between the two proportions. We discuss the theoretical implications of these findings in the General Discussion.

## General discussion

A relatively recent development in the literature on auditory attention in the cocktail party setting is the emergence of a body of research that focuses on instructed switches between voices (e.g., Koch et al., 2011). One of the key issues in this research has been whether preparation during the interval following the voice cue reduces the cost of switching attention from one voice to another. As explained in the Introduction, attempts to resolve this issue have reached somewhat of a conundrum–several studies have revealed numerically modest and inconsistent effects that have not reached statistical significance, whereas a recent study that optimized the voice-switching paradigm specifically with preparation in mind found a substantial and statistically significant reduction in switch cost with preparation (Monsell et al., 2019).

The aim of the present study was to determine what factor(s) are responsible for this inconsistency. Based on previous research, we identified and investigated two such factors–the probability of a switch in the target voice (Experiment 1) and the relative proportion of response-congruent vs. response-incongruent trials (Experiment 2). To the best of our knowledge, the effects of proportion congruent on the reduction in switch cost with preparation have not been examined thus far in either voice switching or task switching. The present study is also the first investigation of switch probability in the cocktail party setting.

## Effects of switch probability

Our RT results reveal clear effects of switch probability. When the probability of a switch of the target voice (gender) was 25%, the switch cost was substantially larger, and its reduction substantially steeper, than when the probability of a switch was high (75%); in the 75%-switch condition, the (smaller) switch cost was still significant, but its reduction with preparation was not. A similar pattern of numerical differences between the two switch probability conditions was present in the error rates, but it did not reach significance. These effects of switch probability can go a long way in explaining the above-mentioned (see also Introduction) discrepancy between the finding of a steep reduction in switch cost with preparation in Monsell et al. (2019), where 33% of trials were switches, and earlier studies which had 50% switches.

What kind of processes may cause the considerable differences in performance between our low versus high switch probability conditions? There are several theoretical accounts of effects of switch probability in the task-switching literature. Most of them can be classified into explanations in terms of sustained cognitive control operating on the scale of minutes to tens of minutes, and explanations in terms of relatively brief engagement of cognitive control, confined to single (or short sequences of) trials, operating on the scale of hundreds of milliseconds to seconds. Henceforth, we will refer to the former accounts as ‘tonic’ and to the latter accounts as ‘phasic.’ The two types of accounts are not mutually exclusive, hence some interpretations of the effects of switch probability have incorporated both (e.g., Siqi-Liu et al., 2022).

The earliest phasic account was proposed by Monsell and Mizon (2006)—who were among the first to document the effect of switch probability on the task switch cost and its reduction with preparation. They suggested that when switches are likely, control processes involved in reconfiguring the task-set may not wait for the task cue, but start earlier. They also suggested two ways in which this may happen—preparing for the task to which a switch is likely, and/or disengaging from/inhibiting the just-executed task. Both of these scenarios should improve performance when the transition turns out to be a task switch, whereas they should worsen performance in the (less likely) case the transition turns out to be a task repetition, thus reducing the difference between switches and repetitions, the switch cost. Evidence that the effects of probability on the switch cost are also observed when there are three tasks in play (Mayr et al., 2013; Siqi-Liu & Egner, 2020) reduces the appeal of the preparation version of Monsell and Mizon’s account, because one would not know which of two alternative task-sets to prepare—but the possibility of a phasic disengagement/inhibition before the task cue remains. A related account is the proposal by Mayr et al. (2013) and Kikumoto et al. (2016) that when the probability of a task switch is low (which means a high probability of a task repetition), cognitive control is applied to maintain the task-set from the previous trial in an active state, but no such effortful task-set maintenance takes place when switches are likely (and repetitions are unlikely). In the context of switches of attention between voices in a multitalker setting, the above accounts would translate into disengaging/inhibiting, or not maintaining across trials, the frequency and prosody profile of the just-listened-to-voice if the probability of switching to another voice is high.

Tonic accounts of the effects of switch probability evolve around the notion that the extent to which cognitive control should be committed to the currently relevant task-set can be characterized along a stability-to-flexibility continuum (e.g., Dreisbach & Fröber, 2019). A low switch probability encourages stability, hence cognitive control should be considerably more committed to the currently relevant task-set than to other potential task-set(s). Conversely, a high switch probability encourages a more flexible processing mode where cognitive control should be only slightly more committed to the relevant task-set than to its alternatives. With regard to the mechanisms that can achieve such adaptive adjustments of the selectivity of cognitive control, Dreisbach and Fröber (2019) have suggested that when switch probability is high this could be realized via loading multiple task-sets into working memory (WM), or via loading a single task-set in WM but lowering the WM updating threshold. Another mechanism that could produce the desired stability–flexibility adaptations in response to switch probability, implemented in a computational model (Musslick & Cohen, 2021), is to modulate how strongly the currently relevant task-set gets activated (relative to the other task-sets in play). This activation can be strong when switch probability is low (but switches are costly), or not as strong when switch probability is high (resulting in less costly switches). In the context of selecting one of two or more simultaneous voices, this would predict a strong activation of the perceptual features of the target voice when an imminent switch is unlikely, but a somewhat weaker activation of the target voice features when one expects to switch to another voice imminently.

The above accounts based on stability–flexibility adaptations also predict that experimental conditions that promote stability (such as conditions with a low switch probability) should result in reduced interference from the responses associated with the competing task-set—and presumably a smaller effect of response (in)congruence (cf. Table 1 in Dreisbach & Fröber, 2019).^5^ Our analysis of the influence of switch probability on the congruence effect did not reveal a detectable interaction, which seems to be consistent with the pattern recently reported by Geddert and Egner (2022). However, the near-null congruence effect for RTs and relatively modest congruence effect for the error rate in the 75%-switch condition left little room for further reduction in the 25%-switch condition. This makes the absence of a detectable influence of switch probability on the congruence effect in our data less conclusive, so that we do not place much weight on this null effect.

Thus, both phasic and tonic accounts of switch probability may account for the present results and further research will be needed to adjudicate between them.

### Effects of the relative proportion of congruent and incongruent trials

Our finding (in Experiment 2) of a substantially (and significantly) larger congruence effect (difference between congruent and incongruent trials) in the 66%-congruent condition than in the 33%-congruent condition for both RTs and errors serves as a ‘manipulation check’ and confirms that the proportion congruent manipulation was effective in eliciting a ramping up in top-down control based on the expected prevalence of conflict (e.g., Bugg & Braver, 2016; Bugg & Crump, 2012). However, our results did not reveal an effect of proportion congruent on the voice switch cost or its reduction with preparation. The significant reduction in switch cost even in the mostly-congruent condition indicates that 1/3 response-incongruent trials (where correct responding requires the selection of the target voice) is sufficient to encourage preparatory top-down control. To interpret these results, we consider the processing stage(s) where the extra selectivity of cognitive control in the 33%-congruent condition could manifest itself.

Since in the voice-switching paradigm the categorization, categories and associated S-R rules are constant, response conflict on an incongruent trial can arise only from the activation of the competing response associated with the number spoken by the nontarget voice. This response conflict can be addressed (at least in part) at an early processing stage (by reducing the perceptual encoding of the nontarget voice) and/or at the later stage of response selection (by ensuring that the response activated by the number spoken by the nontarget voice is not selected). Our results are more consistent with a late locus of resolution of response conflict. As already mentioned, there was no detectable interaction between the proportion congruent manipulation and preparation (alone and in interaction with switching). Moreover, in contrast to some task-switching studies (Meiran et al., 2000; Monsell & Mizon, 2006), where preparation reduced the congruence effect, in our analyses the numerically modest reduction in the congruence effect with preparation did not reach significance. Thus, there is little indication that preparation helped reduce the perceptual encoding of the nontarget voice. It may be that when voice onsets are simultaneous, auditory processing takes time to tune in to the relevant features, hence the nontarget voice’s speech is processed for some time before its processing can be attenuated. This interpretation is supported by a voice-switching study by Nolden et al. (2019), which compared simultaneous with sequential voice onsets, and for the latter, the conditions where the target voice is presented first versus second. They found a smaller congruence effect when the target was presented first than when voices were simultaneous or when the target came second, suggesting more effective suppression of perceptual encoding of the nontarget speech in the target-first condition.

We expected that the condition with a higher conflict prevalence (lower proportion congruent) should result in a smaller switch cost (as indeed reported previously for error rates by Bugg & Braver, 2016, and Schneider, 2015), and potentially in a larger effect of preparation on the switch cost. However, it could also be argued, from the perspective of the stability–flexibility framework, that conditions that encourage selectivity should lead to adjustments towards greater stability, and therefore a larger switch cost (e.g., Dreisbach & Fröber, 2019). The lack of an effect of proportion congruent (see also Geddert & Egner, 2022) may be seen as inconsistent with the notion of a stability–flexibility trade off, or it may indicate that the processes involved in ensuring optimal stability in (or commitment to) a task-set are distinct from processes that monitor for interference specifically after stimulus onset and particularly during response selection.

## Conclusions

The present study has confirmed, in two experiments, that preparation can substantially and significantly reduce the cost of switching auditory attention between simultaneous gender-cued voices. More importantly, we determined one factor that is likely to explain why in most previous voice-switching studies the effects of preparation on switch cost were absent or modest and nonsignificant. This factor is the probability of a voice switch. When switches of the target voice are relatively rare, this results in a large switch cost—which is substantially reduced with preparation. Conversely, when switches are relatively frequent, the switch cost is much smaller and its modulation by preparation is modest or altogether absent. Future research is needed to ascertain whether these effects of switch probability are due to sustained, tonic, adjustments in attentional control and/or to within-trial, phasic, changes in attentional control. We also examined the effect of the relative proportion of response-congruent versus incongruent trials and found that neither the switch cost, nor its reduction with preparation, is substantially modulated by the proportion manipulation, despite a clear indication that this manipulation strongly influenced the control participants exerted over their performance, as indicated by the congruence effect. We conclude that a relatively large proportion of trials where the voices’ utterances require the same response does not discourage preparation following the voice cue.

## Data Availability

The materials used in the study and the data obtained in the study are available for open access in the PsychArchives repository (10.23668/psycharchives.12918).^6^ Neither of the two experiments was preregistered.

## Appendix

### **A.** Experiment 1: Balancing and randomization of numbers spoken by the voices.

The following is an outline of how the script was developed to create sequences which balanced the numbers spoken by the target voice and the nontarget voice in the 25%-switch condition/session (there were structurally equivalent sequences in the 75%-switch condition/session, except that the constraints that applied to switch vs. repeat trials were swapped). For incongruent trials, the eight numbers were equally likely to be spoken by the target voice for all combinations of CSI × switch/repeat × voice gender (each number twice on switch trials and six times on repeat trials). With regard to congruent trials, for 2/3 of the repeat trials the eight numbers were equally likely to be spoken by the target voice for all CSI × voice gender combinations, whereas for the remaining 1/3 of repeat trials and the switch trials this could be achieved only across CSIs—in two CSIs the male voice (when target) spoke numbers 1–4 and the female voice (when target) spoke numbers 6–9, and this was reversed in the other two CSIs. The balancing of the numbers spoken by the nontarget voice was done as follows. For 2/3 of the incongruent repeat trials (32 per CSI per voice), all four possible numbers that could be spoken by the nontarget voice for a given number spoken by the target voice were presented (equally) for all CSI × voice gender combinations. For the remaining 1/3 of the repeat incongruent trials and (separately) for all incongruent switch trials, for each number spoken by the target voice, two of the four possible numbers that could be spoken by the nontarget voice were randomly chosen to be spoken by the female voice (when it was the nontarget), whereas the remaining two numbers were spoken by the male voice (when it was the nontarget); this allocation was done for two CSIs and then it was reversed for the other two CSIs. This ensured that, across all CSIs, on incongruent trials, each number spoken by each target voice (male, female) co-occurred equally with all possible numbers that could be spoken by the nontarget voice. For the congruent trials (the 20% of trials not included in the analysis; see below), the number spoken by the nontarget voice was randomly selected among the three possibilities (excluding the fourth, where the two voices say the same number).

### **B.** Complete inferential statistics for Experiment 1.

**Table:**

Analysis	Effect	*F*	*df*	*p*	η_p_^2^
RT: Switch Prob × Switch/Repeat × CSI	CSI	14.16	3, 93	<.001	.314
	Switch/Repeat	84.64	1, 31	<.001	.732
	Switch Prob	0.17	1, 31	.685	.005
	Switch Prob × Switch/Repeat	16.65	1, 31	<.001	.349
	CSI × Switch/Repeat	3.17	3, 93	.028	.093
	Switch Prob × CSI	0.76	3, 93	.476	.024
	Switch Prob × CSI × Switch/Repeat	7.15	3, 93	<.001	.187
RT 25% switches: Switch/Repeat × CSI	CSI	8.84	3, 93	<.001	.222
	Switch/Repeat	56.24	1, 31	<.001	.645
	CSI × Switch/Repeat	9.35	3, 93	<.001	.232
RT 75% switches: Switch/Repeat × CSI	CSI	7.79	3, 93	<.001	.201
	Switch/Repeat	53.19	1, 31	<.001	.632
	CSI × Switch/Repeat	1.74	3, 93	.171	.053
RT 25% switches & 1400ms CSI: Switch/Repeat	Switch/Repeat	21.92	1, 31	<.001	.414
Errors: Switch Prob × Switch/Repeat × CSI	Switch/Repeat	16.05	1, 31	<.001	.341
	Switch Prob	2.84	1, 31	.102	.084
	CSI	2.43	3, 93	.073	.073
	Switch Prob × Switch/Repeat	2.81	1, 31	.104	.083
	Switch Prob × CSI	1.76	3, 93	.160	.054
	CSI × Switch/Repeat	1.30	3, 93	.279	.040
	Switch Prob × Switch/Repeat × CSI	0.53	3, 93	.649	.017

### **C.** Experiment 2: Balancing and randomization of numbers spoken by the voices.

The numbers spoken by the target voice and the nontarget voice in the 33%-congruent condition were balanced in the trial sequences as follows (there were structurally equivalent sequences in the 66%-congruent condition/session, except that the constraints that applied to congruent vs. incongruent trials were swapped). On congruent trials, all possible combinations of the numbers spoken by the two voices occurred equally (within each participant) for all the combinations of switch/repeat × CSI × target voice gender. On incongruent trials (the number of trials did not permit perfect balancing of all the combinations of the two spoken numbers within each participant), but the balancing was perfect at the level of the numbers spoken by each voice separately. In particular, the eight numbers spoken by the target voice, and (separately) the eight numbers spoken by the nontarget voice occurred equally for all combinations of switch/repeat × CSI × target voice gender. With regard to the combinations of numbers spoken by the two voices on incongruent trials, half of the combinations were perfectly balanced over switch/repeat × CSI × target voice gender. The remaining half of the number combinations were balanced over switch/repeat × target voice gender, but not CSI. In particular, some of the latter number combinations were presented on repeat trials where the female was the target voice and switch trials where the male was the target voice in one CSI, whereas for the other CSI this was reversed. The script also ensured that over groups of 8 participants all number combinations were equally represented over switch/repeat × CSI × target voice gender.

### **D.** Complete inferential statistics for Experiment 2.

**Table:**

Analysis	Effect	*F*	*df*	*p*	η_p_^2^
RT: Cong Prob × CSI × Congruence × Switch/Repeat	Cong Prob	1.52	1, 46	.224	.032
	CSI	54.32	1, 46	<.001	.541
	Congruence	70.61	1, 46	<.001	.606
	Switch/Repeat	235.09	1, 46	<.001	.836
	Cong Prob × CSI	0.76	1, 46	.387	.016
	Cong Prob × Congruence	44.13	1, 46	<.001	.490
	CSI × Congruence	2.48	1, 46	.122	.051
	Cong Prob × Switch/Repeat	2.07	1, 46	.157	.043
	CSI × Switch/Repeat	17.19	1, 46	<.001	.272
	Congruence × Switch/Repeat	14.85	1, 46	<.001	.244
	Cong Prob × CSI × Congruence	1.94	1, 46	.171	.040
	Cong Prob × CSI × Switch/Repeat	0.29	1, 46	.593	.006
	Cong Prob × Congruence × Switch/Repeat	1.56	1, 46	.219	.033
	CSI × Congruence × Switch/Repeat	0.58	1, 46	.451	.012
	Cong Prob × CSI × Congruence × Switch/Repeat	0.01	1, 46	.919	<.001
RT 33% Congruent: CSI × Congruence × Switch/Repeat	CSI	47.07	1, 46	<.001	.506
	Congruence	22.88	1, 46	<.001	.332
	Switch/Repeat	160.91	1, 46	<.001	.778
	CSI × Congruence	0.01	1, 46	.937	<.001
	CSI × Switch/Repeat	13.80	1, 46	<.001	.231
	Congruence × Switch/Repeat	4.94	1, 46	.031	.097
	CSI × Congruence × Switch/Repeat	0.16	1, 46	.688	.004
RT 66% Congruent: CSI × Congruence × Switch/Repeat	CSI	43.50	1, 46	<.001	.486
	Congruence	88.38	1, 46	<.001	.658
	Switch/Repeat	192.97	1, 46	<.001	0.808
	CSI × Congruence	4.22	1, 46	.046	.084
	CSI × Switch/Repeat	13.46	1, 46	<.001	.226
	Congruence × Switch/Repeat	17.59	1, 46	<.001	.277
	CSI × Congruence × Switch/Repeat	0.31	1, 46	.581	.007
Errors: Cong Prob × CSI × Congruence × Switch/Repeat	Cong Prob	9.06	1, 46	.004	.165
	CSI	11.34	1, 46	.002	.198
	Congruence	72.65	1, 46	<.001	.612
	Switch/Repeat	68.00	1, 46	<.001	.596
	Cong Prob × CSI	0.03	1, 46	.858	.001
	Cong Prob × Congruence	23.54	1, 46	<.001	.339
	CSI × Congruence	3.14	1, 46	.083	.064
	Cong Prob × Switch/Repeat	0.16	1, 46	.694	.003
	CSI × Switch/Repeat	6.88	1, 46	.012	.130
	Congruence × Switch/Repeat	29.34	1, 46	<.001	.389
	Cong Prob × CSI × Congruence	2.77	1, 46	.103	.057
	Cong Prob × CSI × Switch/Repeat	0.43	1, 46	.518	.009
	Cong Prob × Congruence × Switch/Repeat	3.67	1, 46	.062	.074
	CSI × Congruence × Switch/Repeat	1.15	1, 46	.289	.024
	Cong Prob × CSI × Congruence × Switch/Repeat	2.72	1, 46	.106	.056

## Footnotes

1 The mathematical expression of probability is a number ≥ 0 and ≤ 1. However, in keeping with the convention of recent task-switching studies that expressed this manipulation in terms of the proportions, we express the manipulation of probability of a switch as a percentage.

2 Monsell et al. (2019) noted two further factors that might have discouraged and/or reduced the effectiveness of preparation in previous voice-switching studies: (1) there was more than one voice per gender, hence the speaker could change even on gender repetition trials; (2) dichotic presentation made the location (side) of the target gender unpredictable. Hence, they presented the voices centrally, (only) one voice per gender.

3 We thank an anonymous reviewer for suggesting this analysis, whose implications are considered in the General Discussion.

4 The exclusion of one participant’s data at the analysis stage has reduced one of the groups to 23, resulting in a slight imbalance (23 vs. 24).

5 We thank an anonymous reviewer for reminding us of this corollary prediction and suggesting the related analysis.
